# Effects of SARS-CoV-2 Infection on Pulmonary Function Tests and Exercise Tolerance

**DOI:** 10.3390/jcm11174936

**Published:** 2022-08-23

**Authors:** Josuel Ora, Bartolomeo Zerillo, Patrizia De Marco, Gian Marco Manzetti, Ilaria De Guido, Luigino Calzetta, Paola Rogliani

**Affiliations:** 1 Division of Respiratory Medicine, University Hospital Policlinico Tor Vergata, 00133 Rome, Italy; josuel78@gmail.com (J.O.); lungbar@gmail.com (B.Z.); paola.rogliani@uniroma2.it (P.R.); 2 Unit of Respiratory Medicine, Department of Experimental Medicine, University of Rome “Tor Vergata”, 00133 Rome, Italy; patrizia.demarco@students.uniroma2.eu (P.D.M.); gianmarcomanzetti@yahoo.it (G.M.M.); ilaria.deguido@students.uniroma2.eu (I.D.G.); 3 Respiratory Disease and Lung Function Unit, Department of Medicine and Surgery, University of Parma, 43126 Parma, Italy

**Keywords:** COVID-19, pulmonary function test, 6MWT

## Abstract

Introduction: Severe acute respiratory syndrome coronavirus 2 (SARS-CoV-2) has been shown to cause long-term pulmonary sequelae. Objects: The aim of this study was to evaluate the consequences of the SARS-CoV-2 infection on pulmonary function and on the 6-min walk test related to the severity of the disease. Methods: A cross-sectional study was conducted at the “Policlinico Tor Vergata” Academic Hospital (Rome, Italy), including 75 patients evaluated in post-COVID clinics at the Respiratory Units between November 2020 and September 2021. Complete pulmonary function tests, 6-min walk tests and persistence of symptoms were performed. Results: Of the 75 subjects, 23 had mild, 16 moderate, 26 severe and 10 very severe COVID-19, classified according to WHO. Very severe patients had a lower FVC (100 ± 10%pr) compared to the other groups (116 ± 16%pr, 116 ± 13%pr, 122 ± 20%pr from mild to severe; *p* < 0.05) and a lower TLC (94 ± 13%pr) compared to the others (102 ± 10%pr, 108 ± 15%pr, 108 ± 12%pr from mild to severe; *p* < 0.05). DLco and DLco/VA were similar among groups. At the 6MWT, distance, rest and nadir SpO_2_ were similar among groups, but all groups presented a significant decrease in SpO_2_ from rest to nadir (Rest SpO_2_: 97.0 ± 1.0% vs. Nadir SpO_2_: 93.6 ± 2.7%, *p* < 0.01). A positive correlation was found between desaturation and delta SpO_2_ (rest—nadir) (R: 0.29, *p* < 0.05) and the Distance Desaturation Product (R: 0.39, *p* < 0.01). Conclusions: These results showed that, although the PFTs are within the normal range, there is still a mild restrictive spirometric pattern after six months in very severe subjects. Moreover, the only persistent pathological sequalae of SARS-CoV-2 infection were a mild desaturation at 6MWT, despite the severity of the infection.

## 1. Introduction

Coronavirus disease 2019 (COVID-19) is caused by severe acute respiratory syndrome coronavirus 2 (SARS-CoV-2) that can cause a large spectrum of clinical presentations from mild illness to a serious disease leading to hospitalization [1].

In the acute phase, the main affected organs are the lungs [2] that can undergo different pathophysiological alterations going from pulmonary consolidation and alveolar epithelium destruction to hyaline membrane formation, capillary damage and bleeding, and alveolar septal fibrous proliferation [2]. Similarly, the radiological and clinical alterations can vary from just minimal opacities with an almost normal chest radiography and mild hypoxemia to very severe disease manifestations with acute respiratory failure and severe hypoxemia [3]. The extensive injury to alveolar epithelial and endothelial cells with secondary fibroproliferation [4] may lead to chronic vascular and alveolar remodeling, causing lung fibrosis and/or pulmonary hypertension [5].

On average, the acute infection can last 2–3 weeks depending on severity of clinical presentation, however, many patients present symptoms that last more than 12 weeks, regardless of disease severity [6].

Some studies have shown abnormal lung function in COVID-19 survivors, characterized by altered lung diffusion capacity of carbon monoxide (DLco), but not DLco/Alveolar Ventilation (VA), and a restrictive ventilatory defect [3,7,8,9]. Lower peripheral oxygen saturation (SpO_2_) at rest and during the 6-min walk test (6MWT), total lung capacity (TLC), airway occlusion pressure after 0.1 s (P0.1) and P0.1/maximal inspiratory pressure (MIP) ratio were found in COVID-19 patients with pneumonia compared to those without pneumonia [7]. However, many studies [7,8,10,11,12] evaluated patients after 1 to 4 months from discharge and little information is provided about what happens in longer periods.

To date, it is not yet clear how long the abnormalities of respiratory function last and whether they have a relationship with the symptoms of post COVID.

For this reason, this study aims to evaluate the consequences of the SARS-CoV-2 infection on pulmonary function and on the 6MWT related to the severity of the disease and the persistence of symptoms.

## 2. Materials and Methods

This was a cross-sectional study performed at the “Policlinico Tor Vergata” university hospital (Rome, Italy) and was conducted according to the STROBE guidelines [13,14] (checklist in the supplement). Severity was defined by the World Health Organization guideline for COVID-19 [15]. Clinical records of the Respiratory Disease Unit’s 87 post-COVID outpatients between November 2020 to October 2021 were reviewed. Patients were recruited if they had a diagnosis of SARS-CoV-2 infection certified by molecular nasal swab and if they performed pulmonary function tests (PFTs) and 6MWT. Twelve patients did not have complete PFTs and were excluded from the study. Of the 75 COVID-19 patients included in the study, 52 had previously been admitted to the Respiratory Disease ward and were classified from moderate to very severe according to the required respiratory support: 16 who needed oxygen were classified as moderate, 26 who needed high flow nasal cannula (HFNC) or non-invasive mechanical ventilation (NIMV) were classified as severe and 10 who needed intensive care unit supplementation were classified as very severe; the other 23 patients who did not use oxygen support were classified as mild.

The investigations were carried out following the rules of the Declaration of Helsinki of 1975, revised in 2013, approved by the ethics committee of the “Policlinico Tor Vergata” (number of trial register 166.22).

## 3. Pulmonary Function Testing

Complete PFTs, including forced vital capacity (FVC) and DL_CO_, determined with the single breath technique (SB), were carried out according to the ATS/ERS guidelines [16,17,18] on a Master Screen Body PFT (Jaeger acquired by Vyaire, Medical, Inc., Mettawa, IL, USA) using European Coal and Steel Community reference spirometric values [19]. Maximal inspiratory pressure (MIP), maximal expiratory pressure (MEP), P0.1 and P0.1/MIP were measured according to the American Thoracic Society/European Respiratory Society (ATS/ERS) statement on respiratory muscle testing [20] using Evans et al. reference value [21].

## 4. 6MWT

The 6MWT was performed in a straight 30 m indoor hallway according to the American Thoracic Society (ATS) guidelines [22]. All patients were tested under standardized conditions by trained operators. The 6MWD was expressed both as absolute value in meters and as %predicted value (%pr), using the Enright and Sherill equations [23]. Heart rate and oxygen saturation were continuously measured at rest (baseline) and during the test until recovery by the Mir Spirodoc oximeter with step counter and accelerometer with VMU motion analysis (Spirodoc, Medical International Research, MIR, Rome, Italy). Thanks to the continuous measurement of these parameters, the saturation time under 90% (T90), the SpO_2rest-nadir_, the Desaturation Distance Ratio (DDR) and the O2-GAP index were computed [24,25,26].

## 5. Statistical Analysis

Statistical analysis was carried out employing Microsoft Excel software. Group comparisons were made using Student’s *t*-test or two-way analysis of variance (ANOVA), as appropriate. A *p*-value < 0.05 was considered statistically significant. Univariate correlations were examined using Pearson’s product moment-correlation. Data are presented as average ± standard deviation (SD) if not differently specified.

## 6. Results

### 6.1. Subjects

Subjects’ characteristics are shown in Table 1. In total, 75 subjects were included in the study: 23 had mild, 16 moderate, 26 severe and 10 very severe COVID, according to the respiratory support they needed. The follow up was six months on average (171 ± 93 days), longer in the moderate group (212 ± 104 days, *p* = 0.032).

Comorbidities are shown in Table 2, and the therapies administered during the COVID-19 are summarized in Table 3.

### 6.2. Pulmonary Function Tests

PFTs are summarized in Table 1. On average, pulmonary function values were within the normal range for all groups, although FVC and TLC were lower in the very severe group (94.0 ± 13.1; *p* = 0.016). No differences were found among groups for DLco, DLco/VA, MIP, MEP, P 0.1/MIP and MVV. The severe group showed a greater P0.1 %pr compared to other groups.

### 6.3. 6MWT

6MWT results are shown in Table 4. No significant differences were observed among groups in the distance traveled, expressed as a percentage of the predicted, in the SpO_2_ at rest and at nadir. No significant differences in T90% and DDR. The distance traveled was on average lower than 100% of the predicted in the very severe group (6MWT distance: 92.0 ± 19.3 m).

**Figure 1:**
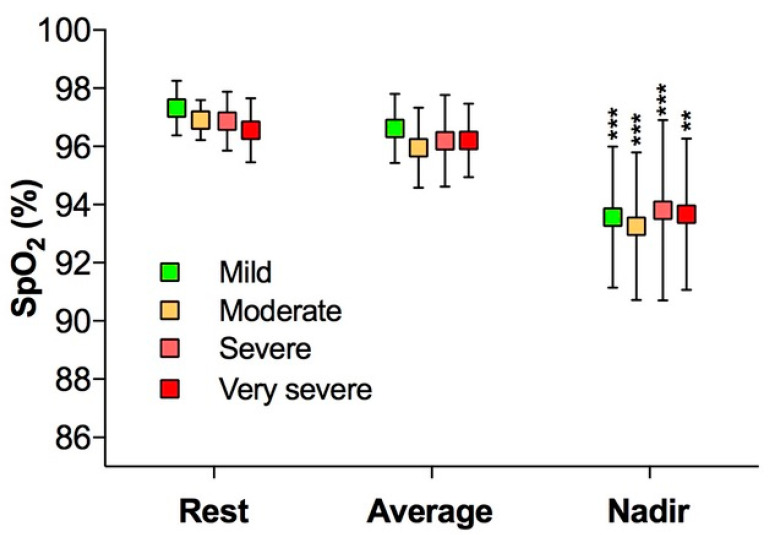
Peripheral saturation at rest (basal), at nadir and the average during the 6-min walk test. Despite the severity, all groups showed a significant desaturation during the test (SpO_2rest-nadir_ **, *p* < 0.01; ***, *p* < 0.001). SpO_2_: Peripheral saturation of oxygen.

In the entire group analyzed as a whole, there was a desaturation during the 6MWT (SpO_2_ rest 97.0 ± 1.0 vs. SpO_2_ nadir 93.6 ± 2.7; *p* < 0.001, Figure 1), the DDR was 0.5 ± 2.3 similar among groups and the O2-GAP was 0 in all groups.

**Figure 2:**
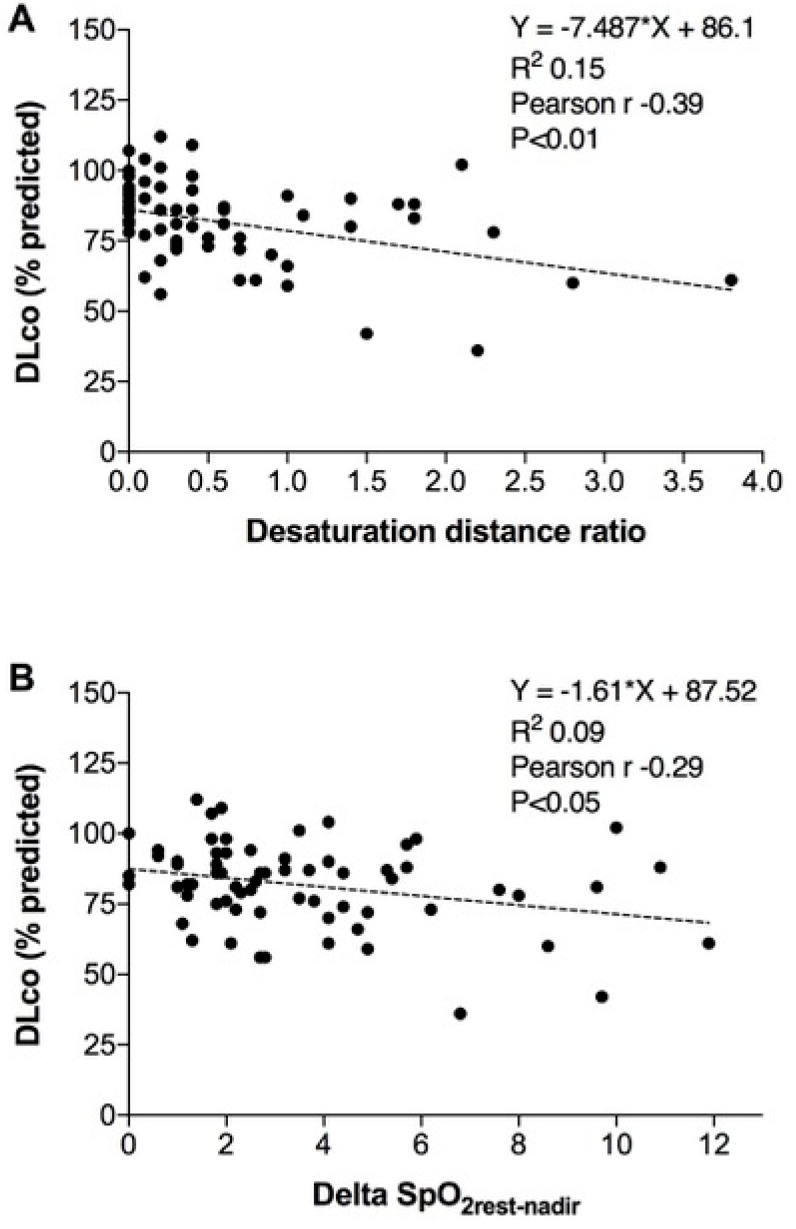
Correlation between the DLco and the DDR (**A**) and correlation between the DLco and SpO_2rest-nadir_ (**B**). DLco–SB: diffusing lung capacity for carbon monoxide; DDR: distance desaturation ratio; SpO_2_: Peripheral saturation of oxygen.

There was a significant correlation between the DLco, %pr and the DDR and the difference between SpO_2rest-nadir_ (*R*² = 0.1487, *p* = 0.001 and *R*² = 0.0861, *p* = 0.015, Figure 2), but not with the DLco/VA, %pr.

### 6.4. Symptom Perception

Fifty-four (72%) subjects reported at least one symptom including dyspnea, fatigue, brain fog, insomnia, anxiety and gastrointestinal discomfort. Symptoms are reported in Table 5. The most common symptoms after six months were dyspnea and fatigue, with a higher prevalence in the very severe group.

The group was divided and analyzed according to the presence or absence of symptoms such as dyspnea, fatigue and brain fog, but no differences in functional parameters or 6MWT variables were found between symptomatic and asymptomatic subjects.

## 7. Discussion

This study mainly shows that after six months of recovery, COVID-19 survivors presented almost normal PFTs, with smaller yet still within normal FVC and TLC only in the more severe subjects. However, a significant desaturation during the 6MWT was present in all the groups, despite the severity of the infection. In this study, no correlations were found between symptoms and any lung function or 6MWT measurements.

Previous studies have shown that in COVID-19 survivors, the main pulmonary function abnormalities were a reduction in DLco with an almost normal DLco/VA and a reduction in FVC and TLC with an increase in the FEV_1_/FVC ratio [2,3,7,8,10,11,12,27].

Mo et al. demonstrated that right after discharge, 47% of patients had a lower DLco and 25% had a lower TLC, and there was a significant difference among the groups according to the severity of the disease [2]. After 30 days from hospital discharge, two other studies [5,28] confirmed the presence of PFT abnormalities in more than 50% of patients characterized by lower TLC, FVC, FEV_1_, and a lower DLco, but a normal DLco/VA. Other studies have investigated lung function after 3-4 months from hospital discharge, still showing a reduction in the DLco in more than 50% of patients, and a reduction in TLC in more than 10% of patients [7,12,29].

Our study showed that PFTs are mostly normal among groups, with a slight difference in patients who had a more severe clinical presentation of the disease, in which FVC and TLC reduction was statistically significant, but clinically within the normal range. Similarly, DLco and DLco/VA were within the normal range without any difference among groups, although the DLco had decreased more than the DLco/VA due to a lower VA. The difference between our results and those in literature can be justified by the longer follow-up and, although some small changes in the PFTs can be detected in the very severe COVID-19 survivors (described as a tendency towards a restrictive pattern, FVC at the lower limits of normality and increased FEV_1_/FVC ratio), they are clinically indistinguishable from a normal patient’s. Our results seem to confirm a complete reversibility of the restrictive pattern, at least in the majority of patients [30].

Our data showed that MIP, MEP and P0.1/MIP were within the normal range in all groups without any difference according to the severity of the disease. This differs from the Anastasio et al. study [7], in which MIP was reduced, mostly in less severe subjects. Authors stated that the decreased MIP and MEP could be due to different factors, such as a virus-induced myopathy affecting respiratory muscles, especially the diaphragm, or could be a possible effect of limited physical activity secondary to the lock down, but this explanation does not justify a more severe impairment in less severe subjects, and it is not what we observed.

Another finding of our study was the desaturation that occurred in all groups during the 6MWT (Table 2, Figure 1 and Figure 2). Three different indices were used to evaluate the desaturation: the SpO_2rest-nadir_, the DDR, and the O2-GAP. The SpO_2rest-nadir_ is a coarse index, it is not related to the intensity of the exercise, it cannot represent the real desaturation during the test and it can be affected by artefacts, mostly during continuous saturation measurement. Conversely, DDR is derived by the desaturation area under the curve divided by the walked distance [24] and is a more reliable physiologic tool to assess pulmonary diseases characterized by involvement of the alveolar-capillary membrane. Instead, the O2-GAP is an index to evaluate the need of oxygen supplementation during exercise [26]. In our population, the O2-GAP was 0, which means that none of our subjects needed oxygen supplementation. The DDR was 0.5 ± 0.8, similar among groups, and it correlates better to DLco than SpO_2rest-nadir_. Although there is no normal DDR value stated in literature, Pimenta et al. [24] reported a normal value of 2.5 (IQ: 2–4.5) compared to pathological values of 11 (9–23) in idiopathic pulmonary fibrosis, while Ijiri et al. [25] reported pathological values between 0 and 0.4 in chronic obstructive pulmonary disease subjects. Our results are closer to those of Ijiri et al., and the correlation with the DLco seems to support gas exchange alterations amplified by exercise. Brown et al. demonstrated with a magnetic resonance-augmented cardiopulmonary exercise testing that the main cause of exercise intolerance in previously hospitalized COVID-19 patients was the inability to increase the stroke volume during exercise, regardless of disease severity [31]. Another study [32] has shown that the exercise limitation in COVID-19 survivors after 3 months from discharge was mainly due to peripheral factors, a reduced oxygen extraction and anemia; for other authors [33], ventilatory inefficiency is the main cause of post COVID exercise limitation. The decreased SpO_2_ demonstrated in our study could be either due to pulmonary membrane abnormalities, which cause ventilatory inefficiency or to peripheral factors, or to a combination of both. Further studies are needed.

Interestingly, we were not able to demonstrate any correlation between symptoms, included dyspnea, and any lung function measurement. Many COVID-19 survivors experience symptoms after the clearance of the acute infection, and this condition is known as long covid [34]. Although several studies have showed some lung function abnormalities, none of them have established a correlation with symptoms. In COVID-19 survivors, many subjects who experience long term dyspnea have no signs of permanent or long-lasting lung damage [35,36]. This also happens for other symptoms. For instance, a cross-sectional study did not find any association between long term fatigue and pro-inflammatory markers [37]. Our studies seem to confirm that long term symptoms in post COVID-19 subjects are complex and multifactorial [34], and single parameters are more useful to determine the severity than the cause of symptoms.

The group of severe patients, but not very severe or moderate ones, showed an increased P0.1. These data could be attributed, in a first hypothesis, to some subjects of this group who presented significantly increased values compared to the average and who may have shifted the significance. It is not clear whether this is just a random datum of no meaning or if it has a pathological meaning that we do not know how to interpret to date.

From a clinical point of view, this study highlights two important aspects: the respiratory function abnormalities can be minimal and, although statistically significant when analyzed in large groups, they are negligible in the evaluation of an individual patient, unlike desaturation during the 6MWT test that is clinically pathological, present among all groups and probably the last post-covid functional abnormalities to heal; moreover, the symptoms of the patients seem to have no correlation with respiratory function and, therefore, the PFR play a role in ruling out the severity of post-covid sequelae rather than in identifying the cause of dyspnea.

The main limitation of this study is that the patients’ pulmonary function tests before the SARS-CoV2 infection were not available, and therefore no comparisons can be made before and after infection; moreover, as a cross-sectional study, the follow-up period is not similar for all the subjects; it was shorter in severe and very severe subjects and this may have overestimated the damage in the most severe patients, although the shortest follow up was in severe patients, while the greatest damage was in very severe patients. Another potential bias in the selection of patients is that not all patients were sent to the post-COVID clinic; admission was determined by the doctors of the department. Moreover, the small number of patients included in each subgroup, especially in the very severe one, could hide some disorders and differences.

## 8. Conclusions

This study showed that after six months of recovery from COVID-19, lung function and diffusion capacity are mostly within normal range; only the most severe subjects showed lower lung volumes and DLco values than those normally observed. The only persistent pathological sequalae of SARS-CoV-2 infection is a mild desaturation at 6MWT, despite the severity of the infection. Furthermore, lung function abnormalities appear to have no correlation with long-lasting symptoms. However, other studies with larger samples are necessary to confirm our results.

## Figures and Tables

**Table 1:** Subjects’ characteristics.

	All Groups (*n* = 75)	Mild (*n* = 23)	Moderate (*n* = 16)	Severe (*n* = 26)	Very Severe (*n* = 10)	*p*-Value
Male, *n* (%)	52 (69.3%)	13 (56.5%)	12 (75%)	19 (73.1%)	8 (80%)	ns
Age, yrs	59.4 ± 11.1	56.8 ± 15.6	60.2 ± 7.0	61.1 ± 9.4	59.7 ± 7.4	ns
Height, cm	171.2 ± 9.6	168.4 ± 9.7	174.6 ± 7.8	170.2 ± 9.6	175 ± 10.4	ns
Weight, kg	81.2 ± 16.8	73.9 ± 16.3	83.0 ± 14.4	84.7 ± 17.3	86.2 ± 16.9	ns
BMI, Kg/m^2^	27.4 ± 4.5	25.7 ± 4.1	27.0 ± 4.6	28.9 ± 4.4	27.8 ± 4.4	ns
mMRC, U	0.5 ± 0.7	0.6 ± 0.7	0.4 ± 0.8	0.3 ± 0.5	1.0 ± 0.6	ns
Follow up, days	171 ± 93	191 ± 92	212 ± 104	134 ± 75	158 ± 95	0.032
FEV_1_, %pr	112.0 ± 17.3	110.0 ± 14.3	115.9 ± 18.8	114.6 ± 19.6	103.2 ± 12.3	ns
FVC, %pr	115.7 ± 16.3	116.0 ± 13.1	121.9 ± 19.6	117.2 ± 15.9	100.3 ± 9.9	0.012
FEV1/FVC, %	78.3 ± 7.9	78.0 ± 10.8	76.6 ± 3.8	78.1 ± 7.1	82.1 ± 5.8	ns
TLC, %pr	104.6 ± 13.0	102.2 ± 9.8	108.4 ± 15.0	108.2 ± 12.3	94.0 ± 13.1	0.016
DLco-sb, %pr	80.3 ± 17.6	81.6 ± 13.5	88.0 ± 10.9	78.5 ± 23.2	71.1 ± 11.7	ns
DLco-sb/VA, %pr	90.9 ± 14.3	90.2 ± 14.3	98.1 ± 16.4	89.2 ± 13.8	86.7 ± 10.9	ns
MIP, %pr	105.8 ± 31.7	97.8 ± 23.4	111.1 ± 30.9	115.2 ± 37.1	89.4 ± 28.3	ns
MEP, %pr	105.3 ± 26.0	101.0 ± 24.6	112.2 ± 26.7	105.2 ± 25.3	104.1 ± 32.4	ns
P0.1, %pr	169.3 ± 73.3	165.9 ± 57.2	138.5 ± 53.1	203.1 ± 93.2	135.4 ± 29.9	0.020
P0.1/MIP, %	143.3 ± 90.1	178.4 ± 102.9	110.3 ± 83.2	144.8 ± 85.0	104.1 ± 39.4	ns
MVV, %pr	109.8 ± 23.7	105.2 ± 15.8	113.7 ± 28.1	116.0 ± 27.3	96.7 ± 15.4	ns

%pr: %predicted value, BMI: Body Mass Index; DLco-SB: diffusing lung capacity for carbon monoxide—single breath; FEV_1_: Forced Expiratory Volume in 1 s; FVC: forced vital capacity; MEP: Maximal Expiratory Pressure; MIP: Maximal Inspiratory Pressure; mMRC: modified Medical Research Council Dyspnea Scale; MVV: Maximal Voluntary Ventilation; ns: not significant; P0.1: airway occlusion pressure in 0.1 s; TLC: total lung capacity; VA: Alveolar Ventilation; Values are expressed as mean ± SD.

**Table 2:** Subjects’ comorbidities and chronic therapies.

	All Groups (*n* = 75)	Mild (*n* = 23)	Moderate (*n* = 16)	Severe (*n* = 26)	Very Severe (*n* = 10)
SAH, (%)	33 (100)	8 (24)	6 (18)	12 (36)	7 (21)
IHD, (%)	12 (100)	4 (33)	1 (8)	5 (42)	2 (17)
Diabetes, (%)	10 (100)	2 (20)	2 (20)	5 (50)	1 (10)
COPD, (%)	3 (100)	2 (67)	0 (0)	1 (33)	0 (0)
Pulmonary Emphysema, (%)	6 (100)	1 (17)	0 (0)	4 (67)	1 (17)
asthma, (%)	3 (100)	2 (67)	0 (0)	1 (33)	0 (0)
OSAS, (%)	4 (100)	2 (50)	0 (0)	0 (0)	2 (50)
CKD, (%)	3 (100)	0 (0)	1 (33)	2 (67)	0 (0)
LAMA, (%)	2 (100)	1 (50)	0 (0)	1 (50)	0 (0)
LABA, (%)	4 (100)	2 (50)	0 (0)	2 (50)	0 (0)
ICS, (%)	2 (100)	1 (50)	0 (0)	1 (50)	0 (0)
ACE-I, (%)	14 (100)	3 (21)	3 (21)	5 (36)	3 (21)

ACE-I: Angiotensin-Converting Enzyme Inhibitors; CKD: Chronic Kidney Disease; COPD: Chronic Obstructive Pulmonary Disease; ICS: Inhaled Corticosteroid; IHD: Ischemic Heart Disease; LABA: Long-Acting β2-Agonist; LAMA: Long-Acting Muscarinic receptor Antagonist; OSAS: Obstructive Sleep Apnea Syndrome; SAH: Systemic Arterial Hypertension.

**Table 3:** Therapies used for SARS-CoV-2 infection.

	All Groups (*n* = 75)	Mild (*n* = 23)	Moderate (*n* = 16)	Severe (*n* = 26)	Very Severe (*n* = 10)
OCS, (%)	56 (100)	12 (21)	13 (23)	24 (43)	7 (13)
Remdesevir, (%)	25 (100)	1 (44)	7 (28)	12 (48)	5 (20)
Tocilizumab, (%)	8 (100)	0 (0)	2 (25)	6 (75)	0 (0)
Ritonavir + lopinavir, (%)	19 (100)	7 (37)	7 (37)	4 (21)	1 (5)
LMWH, (%)	52 (100)	11 (21)	11 (21)	22 (42)	8 (15)
Hydroxychloroquine, (%)	19 (100)	8 (42)	6 (32)	4 (21)	1 (5)
Macrolide, (%)	33 (100)	14 (42)	9 (27)	8 (24)	2 (6)
Azythromicine, (%)	25 (100)	13 (52)	7 (28)	4 (16)	1 (4)
Clarithromicyn, (%)	11 (100)	3 (27)	3 (27)	4 (36)	1 (9)
Ceftriaxone, (%)	24 (100)	6 (25)	5 (21)	10 (42)	3 (13)

LMWH: Low Molecular Weight Heparin; OCS: Oral Corticosteroids.

**Table 4:** Six-minute walk test’s parameters.

	All Groups (*n* = 75)	Mild (*n* = 23)	Moderate (*n* = 16)	Severe (*n* = 26)	Very Severe (*n* = 10)	*p*-Value
6MWTD, %pr	105.3 ± 17.9	106.7 ± 17.8	110.8 ± 18.5	105.4 ± 15.8	92.0 ± 19.3	ns
SpO_2_ rest, %	97.0 ± 1.0	97.3 ± 0.9	96.9 ± 0.7	96.9 ± 0.1	96.6 ± 1.0	ns
SpO_2_ Nadir, %	93.6 ± 2.7	93.6 ± 2.4	93.3 ± 2.5	93.8 ± 3.1	93.7 ± 2.6	ns
T90, %	0.5 ± 2.3	0.1 ± 0.3	0.4 ± 1.1	1.1 ± 3.7	0.0 ± 0.0	ns
HR max, bpm	125.7 ± 15.3	130.4 ± 16.1	123.9 ± 10.5	126.5 ± 16.1	114.7 ± 14.0	ns
SpO_2rest-nadir_	3.3 ± 2.8	3.8 ± 2.4	3.7 ± 2.9	3.1 ± 3.2	2.6 ± 2.5	ns
DDR	0.5 ± 0.8	0.4 ± 0.5	0.7 ± 0.8	0.6 ± 1.0	0.4 ± 0.5	ns
O2-GAP index	0.0 ± 0.0	0.0 ± 0.0	0.0 ± 0.0	0.0 ± 0.0	0.0 ± 0.0	ns

6MWTD: Six-minute walk test distance; DDR: Desaturation distance Ratio; HR: Heart rate; SpO_2_: Peripheral saturation of oxygen; T90: Time of saturation under 90%.

**Table 5:** Reported symptoms at the time of the follow-up.

	All Group (*n* = 75)	Mild (*n* = 23)	Moderate (*n* = 16)	Severe (*n* = 26)	Very Severe (*n* = 10)
Dyspnea, (%)	39 (52)	15 (65)	4 (25)	11 (42)	9 (90)
Fatigue, (%)	40 (53)	14 (61)	6 (38)	13 (33)	7 (70)
Insomnia, (%)	5 (7)	2 (9)	1 (6)	2 (8)	0 (0)
Brain fog, (%)	8 (11)	2 (9)	2 (13)	4 (15)	0 (0)
Gastrointestinal discomfort, (%)	3 (4)	0 (0)	1 (6)	1 (4)	1 (10)
Anxiety/depression, (%)	8 (11)	3 (13)	2 (13)	1 (4)	2 (20)

## Data Availability

The data presented in this study are available on request from the corresponding author.

## Abbreviations

%pr	%predicted value
6MWT	Six Minute Walk Test
ANOVA	One-way Analysis of Variance
COVID-19	Coronavirus Disease 2019
DDR	Desaturation Distance Ratio
DLco-SB	Diffusing Lung Capacity for Carbon Monoxide—Single Breath
FEV1	Forced Expiratory Volume in 1 s
FVC	Forced Vital Capacity
HFNC	High Flow Nasal Cannula
MEP	Maximal Expiratory Pressure
MIP	Maximal Inspiratory Pressure
mMRC	Modified Medical Research Council Dyspnea Scale
MVV	Maximal Voluntary Ventilation
NIMV	Non-invasive Mechanical Ventilation
P0.1	Airway Occlusion Pressure after 0.1 s
PFTs	Pulmonary Function Tests
SARS-CoV-2	Severe Acute Respiratory Syndrome CoronaVirus-2
SpO_2_	Peripheral Saturation of Oxygen
T90	Time of Saturation Under SpO_2_: 90%
TLC	Total Lung Capacity
VA	Alveolar Ventilation
WHO	World Health Organization
